# Behind political affiliation: How moral values, identity politics, and party loyalty have affected COVID-19 vaccination

**DOI:** 10.1371/journal.pone.0330881

**Published:** 2025-09-26

**Authors:** Piergiuseppe Fortunato, Alessio Lombini

**Affiliations:** 1 Department of Economics and Business, University of Neuchâtel, Neuchâtel, Switzerland; 2 Department of Economics, Queen Mary University of London, London, United Kingdom; Villanova University, UNITED STATES OF AMERICA

## Abstract

Falling short of its vaccination goals, the United States faced a critical challenge in ending the pandemic, with political partisanship emerging as a barrier to COVID-19 vaccination uptake. This study investigates the relationship between partisanship, moral values, and vaccination compliance in 3099 US counties during the vaccination campaign, employing descriptive statistics and panel regressions. The findings reveal that the relationship between political partisanship and vaccination uptake varies highly when considering three factors: historical party rootedness, party factions, and co-partisan state governors. We report a widening vaccination rate gap between Republican and Democratic counties, particularly when considering the historical partisanship of a county. Our analysis also reveals that Republican counties with strong support for Trump had vaccination rates that were 2% to 5.9% lower than those observed in Republican counties with low support for Trump. Importantly, moral values significantly mediate the association between partisanship and vaccination compliance. High levels of communal values reduce the predictive power of partisanship and strong support for Trump in explaining vaccination rates by up to 56% and 80%, respectively. The presence of a co-partisan state governor was linked to approximately 3.5% higher vaccination rates in Republican counties and 3.1% higher rates in Democratic-leaning ones. This study demonstrates that the factors explaining vaccine compliance extend beyond dichotomous political orientation. The role of individual moral values is significant in this context. To be most effective, vaccination campaigns—and corresponding messages—should be tailored to reflect the moral and partisanship milieu of their target demographics. The involvement of political leaders, especially in Republican-leaning areas, emerges as a key strategy for increasing vaccine acceptance among these groups.

## Introduction

After the outbreak in the Chinese province of Hubei in December 2019, Coronavirus Disease 2019 (COVID-19) rapidly spread and reached the pandemic status in March 2020. The urgent need to slow viral transmission and reduce mortality jumpstarted a race toward the creation of vaccines, which were created in less than a year after the virus was identified [1]. However, despite strong recommendations and awareness campaigns, acceptance of vaccines targeting SARS-CoV-2 has varied widely between and within countries. In May 2021, a Gallup poll estimated the worldwide unwillingness to take the COVID-19 vaccine (This paper uses the term “COVID-19 vaccination” for simplicity, as it aligns with its widespread use in the scientific literature. However, for technical accuracy, these vaccines actually target SARS-CoV-2, the virus that causes COVID-19.) to around 32 percent or 1.3 billion people [2].

The likelihood of accepting a vaccination can be influenced by several factors [3]. On an individual level, vaccine acceptance and refusal are strongly influenced by varying socioeconomic, cultural, and political contexts. Among these, political orientation plays a crucial role. Indeed, it not only explains the differences in the spread of the disease and the effectiveness of non-pharmaceutical interventions [4,5], but it also shapes how individuals perceive and decide about vaccination [6]. Research in the United States has demonstrated that political affiliation significantly influences community responses to public health initiatives and vaccination efforts [7,8]. Specifically, individuals with conservative orientation tend to exhibit greater risk aversion, which correlates with a lower propensity to participate in vaccination programs for preventable diseases. Conversely, those with liberal views typically show a greater willingness to engage in such programs. The pandemic has further intensified this political divide. Safety guidelines and compliance have varied substantially depending on political orientation, with Republican individuals less likely to follow the Center for Disease Control and Prevention safety guidelines [9,10] and accepting the inoculation of the vaccine [6,11,12]. The influence of partisanship extends beyond the US. For instance, in Germany, party affiliation and political ideology exerted a more significant impact on vaccine attitudes than gender and educational background [13]. Similar trends have also been observed in France [14].

While an extensive body of research has linked partisanship with COVID-19 vaccination compliance, key aspects of this relationship remain unclear. One major gap is the nature of the connection between political views and vaccine acceptance. Questions arise, such as why regions with a specific political orientation consistently show lower vaccination rates and what drives this connection. Furthermore, research on the effect of political partisanship on health policy compliance in the USA reveals a clear divide: Republicans tend to exhibit higher resistance to vaccination and lower adherence to mitigation measures, compared to their Democratic counterparts [15–18]. However, there is limited knowledge regarding the variability—or heterogeneity—of this association. This gap includes several aspects: how the partisanship effect changes with a party rootedness in an area (i.e., for how long a specific area has been affiliated with a party), whether this influence is consistent within the party itself (i.e., do traditional Republicans and “high-Trump-support” Republicans exhibit different attitudes towards vaccination?) and the role political leaders play in shaping vaccination acceptance among their constituents.

Given the complexity of these relationships and the absence of comprehensive theoretical frameworks to fully explain them, we adopt an exploratory approach to address these gaps and deepen our understanding of the connections between political partisanship, individual values, and vaccination rates. We test three hypotheses by employing data from 3099 US counties on presidential elections, vaccination rates against SARS-CoV-2, and survey data on individual moral values. The three hypotheses are outlined as follows, with the first hypothesis addressing whether the association between partisanship and vaccination uptake is heterogeneous.

*Hypothesis 1*: Variation in party rootedness and partisanship intensity is associated with stronger differences in vaccination compliance, while alignment to party factions leads to varying levels of compliance, beyond partisanship.

Here, party rootedness is defined as the duration for which a county has been aligned with a party in presidential elections. While previous research on partisanship and vaccination attitudes have primarily focused on between-party differences [6,9,12,19], we extend this body of knowledge by also examining within-party variation. Specifically, we compare high-Trump-support Republicans with non-high-Trump-support Republicans, focusing on counties with a consistent history of voting Republican. In this context, *“high-Trump-support”* refers to the political ideologies and movements associated with Donald Trump, the 45th US President, and his political base [20–22]. This distinction is important because Trump’s rhetoric on COVID-19 diverged from the core ideology of the Republican Party and research has shown that support for Trump, beyond general Republican partisanship, significantly influenced public skepticism toward COVID-19 and compliance with safety measures [23,24].

Previous studies have highlighted the crucial role of moral values and political affiliation in shaping vaccination behaviors. Indeed, there is evidence of the association between county-level moral values—such as Care, Fairness, Loyalty, Authority, and Purity—and vaccination rates in the United States and the United Kingdom [25,26]. Similarly, regions prioritizing respect for the traditions and their community (communal values) exhibit lower vaccination rates, while those emphasizing fairness and care for the vulnerable (universalist values) show higher compliance. However, a comprehensive understanding of how these two dimensions of moral values interact with partisanship to shape vaccination behaviors is still lacking. Addressing this question can help in developing targeted intervention strategies and enhancing our understanding of vaccine acceptance and behavior across social groups. We investigate this through *hypothesis 2*.

*Hypothesis 2*: Moral values mediate the association between partisanship and vaccination compliance.

To test this hypothesis, we draw on previous work on Moral Foundation Theory (MFT) to measure the importance of a broad spectrum of values and study what factors can explain the connection between partisanship and vaccination rates in the US [26–28]. Specifically, we expand on prior research that developed a framework contrasting universalist and communal moral values. (This division has also been referred to as the universalism-particularism cleavage [29].) Universalist values are based on the notion of care for others and ideas relating to equality, justice, and rights, which therefore favor bridging between different groups. In contrast, communal values relate to more bonding values based on in-group loyalty and respect for authority, which strengthens ties within specific communities or relationships. We diverge from the traditional binding vs. individualizing distinction in MFT, opting instead for the universalist vs. communal cleavage, which excludes the Purity/Sanctity dimension. Although previous studies have shown that Purity/Sanctity negatively correlates with vaccination rates [26,28], we opted for the communal versus universalist cleavage, as it is more thoroughly integrated into research on political partisanship [29–31]. For instance, this division has been embedded in a conceptual political model that examines both the supply and demand sides of morality in voting contexts [30]. Therefore, this framework aligns more directly with our aim of exploring political influences on moral values and vaccination behavior.

Lastly, we study a third dimension of political influence: the role of political leaders who share the same political orientation (*co-partisanship effect*). Compliance with voluntary or legally enforced public policies often requires individuals to sacrifice some personal autonomy in favor of state directives [32]. For citizens to accept this trade-off, they must trust that the policy is beneficial [17], and this trust is generally stronger when the political leader is a co-partisan [33,34]. During the US COVID-19 vaccination campaign, state governors expressed mixed attitudes toward vaccinations, but overall supported the importance of getting vaccinated. (Specifically, in 2021 alone, 41 states, representing approximately 81% of the U.S. population, implemented either a state-level COVID-19 vaccine incentive program or a non-incentive mitigation policy [35].) While this was more evident among Democrats, Republican governors defended the right to refuse the vaccine while still urging hesitant residents to get vaccinated [35,36]. Notably, all US state governors were vaccinated early in the campaign [37]. This is particularly relevant, as the literature highlights the tendency for citizens to mimic the actions of co-partisan elites as one of the key mechanisms of the co-partisan effect [17]. Overall, findings within the literature indicate a positive effect of co-partisanship on compliance with health policies. Investigations into vaccination rates among partisans during the Bush and Obama administrations have uncovered a higher likelihood of vaccination, by 4–10 percentage points, among those aligned with the sitting president’s party [34]. Furthermore, research suggests that the discrepancy in compliance between counties with Republican and Democratic leanings narrows by approximately 5%–7% when stay-at-home orders are issued by Republican governors, as opposed to Democratic ones [17]. We expand this body of evidence by investigating the effect of co-partisanship on vaccination compliance against the SARS-CoV-2 virus and its interaction with moral values. Given that communal values emphasize respect for authority and loyalty to one’s community (such as family or country), we hypothesize that the co-partisanship effect will be more pronounced in areas with strong communal values.

*Hypothesis 3*: People are more inclined to comply with COVID-19 vaccination mandates when issued by a co-partisan. This tendency is amplified in regions with a strong presence of communal values.

Our results complement and strengthen the existing literature in several respects. We show that historical partisanship is strongly associated with variations in vaccination uptakes, with differences as large as 12.7 percentage points. We also find significant differences within the Republican constituency by showing that high-Trump-support counties complied up to 5.9 percent less with vaccination mandates compared to other Republican-leaning counties. In exploring the underlying factors of the connection between political preferences and vaccination, we find that a significant part of the effect of political partisanship on vaccination rates fades away when we account for moral values. In particular, when we restrict the analysis to communities characterized by high communal values, the effect of partisanship on vaccination compliance is approximately up to 55.7% weaker compared to the baseline. Finally, we highlight the role of co-partisanship for vaccination compliance, showing that counties comply, on average, over three percentage points more with vaccination mandates when the governor of the state is affiliated with the party that won the county in the presidential election. In turn, this shows how Americans’ acceptance of vaccination changes based on who is in power.

## Materials and methods

### Data

For this study, we employ data at the county level obtained from different sources. First, the Centers for Disease Control and Prevention (CDC) provides daily county-level data on cumulative COVID-19 vaccination rates starting from late December 2020 [38]. As the main dependent variable, we consider the percentage of fully vaccinated people (i.e., individuals who have had the second dose of a two-dose vaccine or one dose of a single-dose vaccine) based on the jurisdiction and county where the recipient lives. We use data for 3099 counties from January 2021 to May 2022.

Second, the Massachusetts Institute of Technology (MIT) Election Data and Science Lab provides data at the county level on the total votes collected by each political party in the US presidential election held between 2000 and 2020 [39]. We use these data to construct several measures of political partisanship, which are alternatively used in our analyses. To measure the “political rootedness” of a county we use *rooted partisanship*. To measure the “intensity” of partisanship in 2020 we create a categorical variable labeled as *partisanship (in 2020)*. To distinguish the effect of factions in the Republican Party (i.e., high-Trump-support Republicans versus non-high-Trump-support Republicans), we created a dummy variable identifying counties historically leaning Republican but favoring Trump over other traditional Republican candidates. We call this variable *High-Trump-Support*. Finally, following the approach often adopted in the literature, we also create a simple dummy variable labeled as *Republican county*. (Several studies consider the share of Republican votes as an alternative measure [40]. However, to simplify the comparison of the coefficients with our variables of interest, we opted to employ a dichotomous variable as our benchmark instead.) A detailed description of the partisanship variables is given in Table 1.

**Table 1:** Partisanship variables.

Variable	Values (Reference Category)	Description	Number of Counties
*Rooted Partisanship*	Safe Democratic (RC)	The Democratic Party always won the county in presidential elections from 2008 to 2020	448
	Safe Republican	The Republican Party always won the county from 2008 to 2020	2193
	Swing	Each of the two parties won the county at least once	458
*Partisanship (2020)*	Strongly Democratic (RC)	The Democrats won the county by more than 10 percentage points in 2020	456
	Slightly Democratic	The Democrats won the county by less than 10 percentage points in 2020	72
	Strongly Republican	The Republicans won the county by more than 10 percentage points in 2020	2497
	Slightly Republican	The Republicans won the county by less than 10 percentage points in 2020	74
*High-Trump-Support*	1	The vote shares for the Republican Party when Trump was a candidate in presidential elections (2016 and 2020) were higher than those in the years 2000 to 2012 and the Republican Party achieved the majority of votes in the county in both 2016 and 2020 presidential elections	1507
	0 (RC)	Otherwise	686
*Republican County*	1	The Republican Party obtained at least 50%+1 votes in the county	2542
	0 (RC)	Otherwise	557
*Co-partisanship*	1	The Republican (Democratic) Party always won the county from 2008 to 2020 and the state governor is affiliated with the Republican (Democratic) Party	1555
	0 (RC)	Otherwise	1183

Notes: Variables values are constructed by referring to presidential elections in the United States. *RC* indicates the reference category used for each of the four categorical variables.

Third, we draw on previous work on Moral Foundation Theory (MFT) to measure the importance of a broad spectrum of values and study what factors can explain the association between partisanship and vaccination rates in the US [27,28]. These seminal studies propose a new positive framework of morality, that is, the MFT that opposes universalist to communal moral values. Universalist values are based on the notion of care for others and ideas relating to equality, justice, and rights, and that therefore favors bridging between different groups. In contrast, communal values relate to more bonding values based on in-group loyalty and respect for authority, which strengthens ties within specific communities or relationships. In this study, we use data on the value scores at the county level for the period 2015-2018 to construct a variable that measures the relative importance of communal versus universalist moral values as the simple difference between the scores of each category. (S1 Text in the Supporting Information provides a more detailed description of MFT and how our measures for moral values are built.) To incorporate social factors influencing vaccination attitudes [17], we use data and the definition of social capital in 2017 as a proxy for social trust [41]. The specific variable that we use is an index with several standard components (electoral turnout; response rate to the 2010 census; violent crimes; non-profits per capita; religious congregations per capita; and a family unity sub-index), which is provided by the US Congress Joint Economic Committee [42]. Other than using the continuous version of the variable, we also create a dummy variable taking value 1 if the Social Capital index associated with a specific county has a value in the lower quartile of the distribution and 0 otherwise.

Finally, we also collect data for standard predictor variables. The daily cumulative rates of COVID-19 cases and deaths per 1,000 people are provided by the New York Times [43]. Socio-economic data on educational attainments, unemployment, and per capita income come from the Economic Research Service of the US Department of Agriculture [44]. We gather county-level information on demographic and population characteristics such as age structure, race, household size, exposure to the internet, population density, and health insurance coverage from the US Census Bureau data [45]. Data on hospital beds per 1000 people and intensive care unit (ICU) beds are sourced from the Healthcare Cost Report Information System (HCRIS) and an open hospital facilities dataset produced by Definitive Healthcare [46]. All datasets were merged using the five-digit County Federal Information Processing Standard (FIPS) code identifiers. S1 Table in the Supporting Information shows the summary statistics for the variables used in this study.

### Model specification

To test our hypotheses, we employ a threefold empirical strategy. (All the regressions have been run with the Stata command *reghdfe* unless otherwise specified.) For *hypothesis 1*, we conduct four distinct panel regressions to analyze and compare the relationship between COVID-19 vaccination rates and political partisanship across counties, using the four political partisanship variables outlined above. Specifically, we calculate the baseline equation as follows:

$$\gamma_{it}=\;\alpha +\beta_{1}partisanship\;measure_{i}+\beta_{2}(partisanship\;measure_{i}\;\times \;quarter_{q})\;+\beta_{3}cases_{it-7}+\beta_{4}deaths_{it-7}+\theta_{i}+p_{m}+state_{s}+state_{s}\;\times \;p_{m}+\epsilon_{it}$$ (1)

where $\gamma_{it}$ is the cumulative percent of fully vaccinated people (individuals inoculated with the second dose of a two-dose vaccine or with one dose of a single-dose vaccine) for a given county *i* on day *t*. *partisanship measure* is one among our four categorical measures of partisanship (i.e., *rooted partisanship*, *partisanship (2020)*, *Republican county*, and *High-Trump-Support*. *quarter*_*q*_ is a vector of dummy variables indicating quarters from Q1 2021 to Q2 2022 (where Q2 2022 includes only April and May). *cases*_*it*−7_ and *deaths*_*it*−7_ are respectively the daily cumulative rate of COVID-19 cases and deaths per 1,000 people at the county level (lagged by one week). Our main coefficient of interest is *β*_2_ on the interaction between rooted $partisaship\;measure_{i}$ and *quarters*_*q*_. It captures the differential evolution of vaccination rates in counties with different vote preferences, as a proxy for political partisanship, throughout the vaccination campaign. $\theta_{i}$ is a vector of county-level predictor variables. (Health and economic controls include the unemployment rate, poverty rate, median income, proportion of people with less than a high school diploma, population density, proportion of people with internet subscription, the proportion of people older than 65 years old, proportion of minority, the proportion of people with health insurance, and the number of hospital beds of all types per 1000 people.) The specification also includes a rich set of fixed effects. *p*_*m*_ is a set of month-fixed effects that account for common time trends such as changes in federal policies and in the information available to all citizens affecting the common evolution of vaccination. The state-fixed effects *state*_*s*_ absorb all differences in the vaccination measure across the state due to time-invariant characteristics. To further strengthen the identification, we include state-per-month fixed effects. This allows us to control for possible non-linear time trends specific to each state, capturing monthly state variation in vaccination policies through the sample period. *α* is the constant term and $\epsilon_{it}$ is the stochastic error term. (The model is estimated by Ordinary-Least Squares (OLS) linear regression, and all estimations are conducted with Driscoll and Kraay standard errors [47,48] or two-way clustering [49] to account for general forms of cross-sectional and temporal dependence.)

As the second part of our research, we test *hypothesis 2* by introducing in the analysis a relative measure of communal and universalist values. In particular, we first regress the share of fully vaccinated individuals on moral values and social capital. This allows us to evaluate whether moral values are associated with vaccination compliance. Then, we replicate the baseline model on two distinct subsamples of our dataset: one where we include only counties with low levels of universalist values and one where we include counties with only low levels of social capital. This approach enables us to examine if the effect of partisanship varies for fixed levels of moral values.

Finally, we test *hypothesis 3* by examining the effect of co-partisan politicians on COVID-19 vaccinations. This involves assessing whether individuals are more inclined to comply with vaccination directives when they are shared by a governor of the same political party, and if this “co-partisanship effect” varies depending on the community’s level of communal values. Table 1 details the operationalization of co-partisanship in our study. In testing *hypothesis 3*, we formally estimate the following equation:

$$\gamma_{it}=\;\alpha +\beta_{1}copartisanship_{it}+\beta_{2}communal\;values_{i}\;+\beta_{3}(copartisanship_{it}\;\times \;communal\;values_{i})\;+\beta_{4}cases_{it-7}+\beta_{5}deaths_{it-7}+\theta_{i}+p_{m}+state_{s}+state_{s}\;\times \;p_{m}+\epsilon_{it}$$ (2)

where all the terms are identical to those of Eq. 1 except for the inclusion of *copartisanship*_*it*_, which is a dummy variable that equals one if the state governor and the most-voted presidential candidate in 2020 are from the same party. Additionally, we explore whether the effect of co-partisanship on vaccination compliance depends on the level of communal values. This exercise is motivated by communal values being related to respect for authority and loyalty to the “in-group”, which may amplify the co-partisanship effect. To investigate this relationship, we include in the regression an interaction term between co-partisanship and the index measuring communal values.

Two comments are useful. First, from a statistical perspective, we account for monthly changes in vaccination policy and information at both the state and federal levels by including state-by-month and month-fixed effects in all regression models. These fixed effects absorb time-varying factors common to all counties within a state or nationwide—such as new health policies or public health communications—thus isolating within-state variation in vaccination rates (see [50] for a discussion of this method). Notably, employing fixed effects to control for both observable and unobservable factors is standard practice in studies examining COVID-19 outcomes and partisanship [9,51–53]. Second, our analyses do not allow for causal conclusions. Establishing causality between political partisanship and vaccination attitudes is inherently challenging given the nature of political beliefs, a limitation widely acknowledged in the literature. Accordingly, we follow the approach of similar studies by using linear regression to test the relationships we hypothesised between political partisanship, vaccination behaviour, and moral values [6,12,19,40,54–56].

## Results

### Political partisanship and COVID-19 vaccination rates

**Fig 1:**
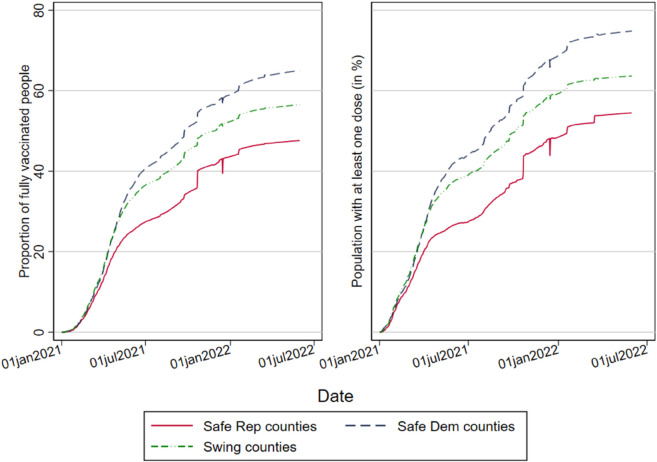
Daily proportion of fully vaccinated people and people who received at least one dose by 2000-2020 US party partisanship (by rooted partisanship values). Counties are identified as either Safe Democrat or Safe Republican if the party won all 2008-2020 presidential elections. Counties are classified as Swing when neither the Democratic nor the Republican parties have consistently won all presidential elections between 2008 and 2020.

To illustrate the relationship between political partisanship and vaccination attitudes, we plot our preferred measures of political partisanship against vaccination rates in US counties. Fig 1 shows the daily changes in the vaccination status of US counties based on the *rooted partisanship* variable, which uses data on the last four presidential elections to classify counties as either “safe Democratic” or “safe Republican”, with the former represented in blue and the latter in red. Additionally, a green line depicts “Swing” counties. We find that safe Democratic counties consistently had a higher proportion of fully vaccinated people and those who received at least one dose, compared to safe Republican counties. This trend persisted throughout the observation period, with the gap widening further after spring 2021, despite Biden’s announcement of a COVID-19 action plan that mandated vaccination for a significant portion of the American workforce. (Refer to S1 Fig in the Supporting information for a timeline of key events related to the COVID-19 vaccination campaign in the US.) Interestingly, we also observed that the share of vaccinated individuals in swing counties was located between the extremes of the safe Democratic and Republican counties.

**Fig 2:**
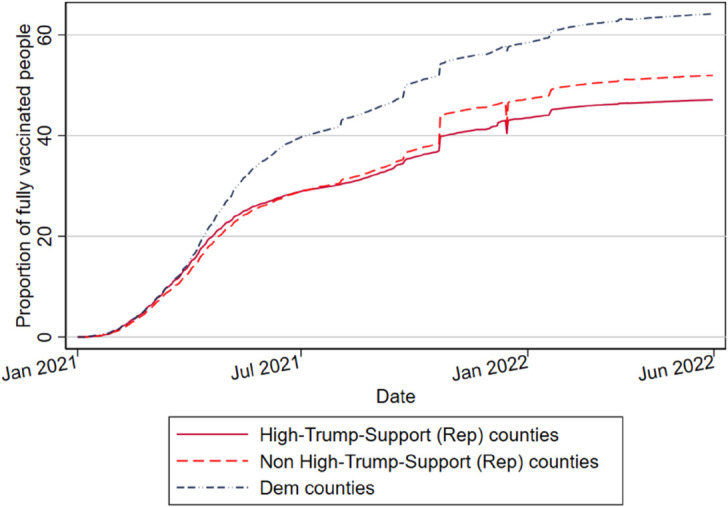
Daily proportion of fully vaccinated people across Republican-leaning counties. The figure displays the proportion of fully vaccinated people across U.S. counties differentiated by political partisanship. Republican counties are identified as *“high-Trump-support”* if the vote shares for the Republican Party when Trump was a candidate in presidential elections (that is, in both 2016 and 2020) were higher than those in the years 2000, 2004, 2008, and 2012 and if the Republican Party achieved the majority of votes in the county in both the 2016 and 2020 presidential elections. Republican counties are defined as Non “high-Trump-support” otherwise.

Fig 1 demonstrates that heterogeneity in vaccination responses can be attributed not only to partisanship itself but also to its historical rootedness. We build upon this preliminary evidence by showing the existence of strong within-party differences in attitudes towards vaccinations. In particular, Fig 2 shows that high-Trump-support counties recorded vaccination rates up to around 5 percentage points lower than Traditional Republicans. Interestingly, the divide around vaccination grew when the spread of the disease (and the fear of the virus) slowed from its hateful boil during the second half of 2021. Figs 1 and 2 suggest that political preferences, both between and within parties, may significantly influence vaccination behavior. However, these patterns could also be affected by confounding factors.

Table 2 shows the regression results obtained from regressing vaccination rates on political partisanship measures, after controlling for health and socio-economic confounding variables (Eq 1). (All reported coefficients in tables presenting regression estimations are unstandardized unless otherwise specified.) The findings reveal that partisanship with the Republican Party correlates negatively with vaccination compliance. Most importantly, the strength of this negative correlation differs depending on the specific political variables analyzed. When we simply consider partisanship in binary terms (Model 1), counties that favored the Republican candidate in the 2020 presidential election had, on average, between −4.3% and −11.3% fewer vaccinated people than in counties that favored the Democrat candidate. Relative to this benchmark measure, when we take into account the historical rootedness of partisanship (i.e. when we differentiate counties where Republican/Democrat presidential candidates consistently had the majority of votes over multiple elections), we see that this gap widens, ranging now between −4.8% and −12.7% (Model 3). A similar conclusion can be drawn when we consider the intensity of partisanship in the 2020 presidential election (Model 2). Finally, Model 4 indicates the existence of relevant intra-party differences in vaccination uptake that are linked to leader preferences. More particularly, Republican counties that preferred Trump to past candidates, have faced between −2% and −5.9% fewer vaccinated people compared to other Republican counties.

**Table 2:** Political partisanship and COVID-19 vaccination rates.

Variable	Model (1)	Model (2)		Model (3)	Model (4)
Partisanship measure	Republican county	Partisanship (in 2020)		Rooted partisanship (Safe Rep)	High-Trump Support
		(Slightly Rep)	(Strongly Rep)		
Main effects	2.732***	1.307***	2.647***	2.383***	2.133***
	(0.473)	(0.434)	(0.541)	(0.536)	(0.429)
Partisanship measure × quarter					
× Q2 2021	−4.351***	−2.349***	−4.887***	−4.806***	−2.060***
	(0.745)	(0.702)	(0.858)	(0.842)	(0.489)
× Q3 2021	−7.980***	−4.473***	−9.055***	−8.803***	−4.021***
	(0.845)	(0.909)	(1.005)	(1.029)	(0.675)
× Q4 2021	−9.726***	−5.049***	−11.079***	−10.896***	−5.079***
	(0.865)	(0.963)	(1.020)	(1.070)	(0.497)
× Q1 2022	−10.918***	−5.193***	−12.414***	−12.185***	−5.632***
	(1.083)	(1.077)	(1.286)	(1.349)	(0.524)
× Q2 2022	−11.308***	−5.317***	−12.901***	−12.730***	−5.855***
	(1.138)	(1.049)	(1.339)	(1.390)	(0.537)
Constant	−342.186	−340.563		−112.806	−2680.951***
	(1035.912)	(1046.861)		(1058.111)	(734.083)
Observations	1259727	1259727		1259727	1010850
R-squared	0.888	0.889		0.890	0.892
Controls	Yes	Yes		Yes	Yes

Notes: The dependent variable is Fully vaccinated people (%). Models 1-4 include state, month, and state*month fixed effects. Model 4 is conducted only on counties where Republicans obtained the majority of votes in the 2020 presidential election. Health and economic controls include: unemployment rate, poverty rate, median income, proportion of people with less than HS diploma, population density, proportion of people with internet subscription, proportion of people older than 65 years old, proportion of minority, proportion of people with health insurance, and number of hospital beds of all types per 1000 people. Coefficients of interaction terms “Slightly Dem x Quarter” and “Swing x Quarter” are omitted for brevity. Q1, Q2, Q3, and Q4 refer to the periods: Jan 1–Mar 31, Apr 1–Jun 30, Jul 1–Sep 30, and Oct 1–Dec 31, respectively. All models use two-way robust standard errors clustered by state and week. Robust standard errors are in parentheses. *** *p* < 0.01, ** *p* < 0.05, * *p* < 0.1.

These results confirm *hypothesis 1*, showing that the association between political orientation and vaccination compliance is highly heterogeneous. In particular, they suggest that the intensity of partisanship and party rootedness are predictors of substantial differences in vaccination compliance. Moreover, the findings show that compliance varies significantly even within the same party, with vaccination rates among Republican-leaning counties differing by up to 5.8 percentage points depending on support for specific political leaders.

### The role of moral values and partisanship

In *hypothesis 2*, we argue that moral values mediate the relationship between partisanship and vaccination intake. This hypothesis is tested by exploring whether individual communal values explain part of the effect of partisanship on COVID-19 vaccination compliance. Given the substantial evidence pointing to the critical role of social factors in this context [40,57,58], we also assess the degree to which social capital—community networks and norms—mediates the political partisanship’s effects on vaccination compliance. Following this, we juxtapose this impact against the influence of individual moral values to evaluate how individual moral values and social capital collectively influence vaccination decisions.

We begin the analysis by regressing the share of vaccinated people on our measures of moral values and social capital (S2 Table). Counties characterized by a high relative diffusion of communal values (Model 1) display on average lower vaccination rates in all the quarters examined in the study. In contrast, counties characterized by high levels of social capital display higher vaccination compliance (Model 3). However, this effect is statistically significant and increasing over time only when we include the relative importance of communal values vs. universalist values. Overall, the results from the S2 Table provide evidence of a positive relationship between universalist values, as well as social capital, and vaccination compliance in US counties.

**Fig 3:**
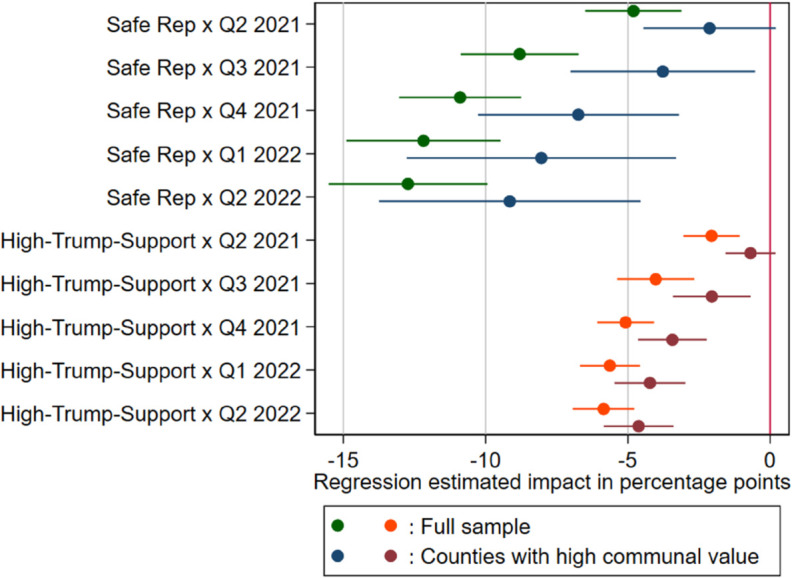
Effect of rooted partisanship and high-Trump-support on vaccination rate compliance. The graph displays the difference over time in vaccination compliance between rooted Democratic and rooted Republican counties (green and blue plots) and between high-Trump-support Republican and Traditional-Republican counties (orange and red plots). Q1, Q2, Q3, and Q4 refer to the periods: Jan 1–Mar 31, Apr 1–Jun 30, Jul 1–Sep 30, and Oct 1–Dec 31, respectively.

In Table 3, we formally test *hypothesis 2* by comparing how partisanship correlates with vaccination rates at different levels of communal values and social capital. Specifically, we replicate the baseline regressions 3 and 4 from Table 2, but this time, we narrow our analysis to counties that fall into two categories: counties in the upper quartile of the distribution of relative communal values (Models 1 and 2) and counties with social capital values below the mean (Models 3 and 4). Fig 3 shows the change in the partisanship effect over time.

**Table 3:** Impact of political partisanship on vaccination rate for communities with low levels of universalist values and social capital.

Variable	Sample of counties			
Partisanship measure	Upper quartile of relative communal values relative communal values		Below-the-mean values of Social Capital Index	
	Model (1)	Model (2)	Model (3)	Model (4)
Swing × Q2 2021	0.602		−1.824**	
	(1.093)		(0.823)	
Swing × Q3 2021	−0.180		−3.664***	
	(1.460)		(1.041)	
Swing × Q4 2021	−1.909		−4.855***	
	(1.624)		(0.985)	
Swing × Q1 2022	−2.405		−5.474***	
	(2.093)		(1.132)	
Swing × Q2 2022	−3.213		−5.927***	
	(1.948)		(1.078)	
Safe Rep × Q2 2021	−2.127*		−4.290***	
	(1.153)		(0.848)	
Safe Rep × Q3 2021	−3.773**		−7.613***	
	(1.609)		(1.144)	
Safe Rep × Q4 2021	−6.740***		−9.838***	
	(1.750)		(1.201)	
Safe Rep × Q1 2022	−8.042***		−11.244***	
	(2.346)		(1.606)	
Safe Rep × Q2 2022	−9.151***		−11.862***	
	(2.280)		(1.693)	
High-Trump-Support× Q2 2021		−0.689		−1.726***
		(0.435)		(0.532)
High-Trump-Support× Q3 2021		−2.049***		−3.349***
		(0.675)		(0.866)
High-Trump-Support× Q4 2021		−3.438***		−4.331***
		(0.597)		(0.615)
High-Trump-Support× Q1 2022		−4.224***		−4.759***
		(0.617)		(0.741)
High-Trump-Support× Q2 2022		−4.620***		−4.914***
		(0.607)		(0.786)
Constant	−994.669	1211.114	−3267.677***	−3071.745**
	(1196.433)	(1234.267)	(906.783)	(1241.903)
Observations	590400	631637	537989	483542
R-squared	0.869	0.894	0.878	0.897

Notes: The dependent variable is Fully vaccinated people (%). Analysis in models 1 and 2 are run over the upper quartile of relative communal values, while analyses in models 3 and 4 are run over counties with below-the-mean values of Social Capital Index. Models 1-4 include state, month and state*month fixed effects. Models 2 and 4 are conducted only on counties where Republicans obtained the majority of votes in the 2020 presidential election. The regression models include the main effects of the interaction terms between partisanship measures and quarters, though they are omitted from the table for brevity. All models include the following health and economic controls: total COVID-19 case rate per 1000 people, total COVID-19 death rate per 1000 people, unemployment rate, poverty rate, median income, proportion of people with less than HS diploma, population density, proportion of people with internet subscription, proportion of people older than 65 years old, proportion of minority, proportion of people with health insurance, and number of hospital beds of all types per 1000 people. Q1, Q2, Q3, and Q4 refer to the periods: Jan 1–Mar 31, Apr 1–Jun 30, Jul 1–Sep 30, and Oct 1–Dec 31, respectively. All models use two-way robust standard errors clustered by state and week. Robust standard errors are in parentheses. *** *p* < 0.01, ** *p* < 0.05,eak * *p* < 0.1.

This approach allows us to estimate the percent changes in the effect of partisan rootedness and leader preference (compared to the baseline model) when we look at specific ranges of moral values and social capital. When we restrict the analysis to communities characterized by high communal values, the effects of Republican-rooted partisanship and high-Trump-support on vaccination compliance are approximately up to 55.7% and 80 % weaker compared to those of the baseline, while they are both approximately up to 13.5% and 14.5% weaker when we look at counties with low social capital. Our findings reveal the existence of an interplay between political preferences, moral values, and social capital in relation to vaccination acceptance. While both moral values and social capital serve as partial mediators of this effect, the results indicate that individual values exert a greater influence than collective factors. These results confirm our *hypothesis 2*, revealing the role of individual values in shaping vaccination attitudes.

### Co-partisanship in times of COVID-19

**Fig 4:**
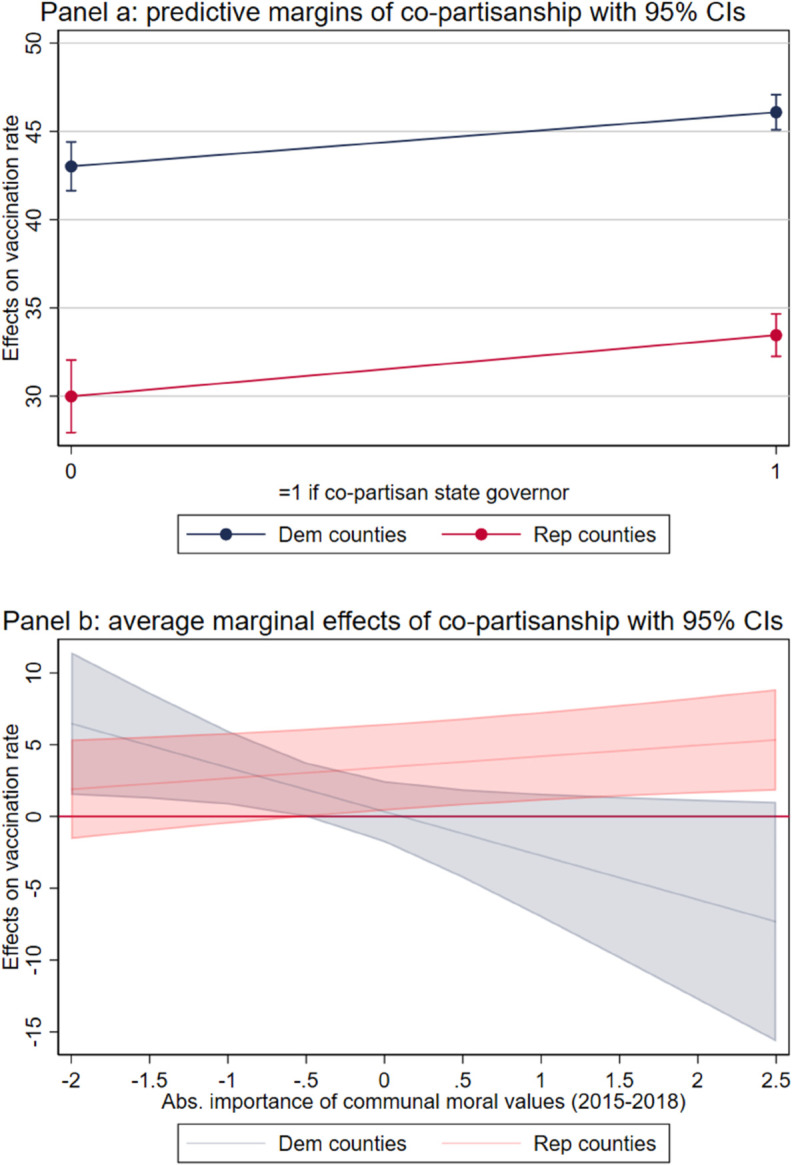
Co-partisanship, vaccination compliance and moral values. Panel a) displays the predictive margins of co-partisanship across Democratic and Republican counties separately. Panel b) displays the average marginal effects of co-partisanship at different levels of moral values by Democratic and Republican counties separately.

*Hypothesis 3* posits the existence of a “co-partisanship” effect. This effect suggests that individuals are more inclined to comply with vaccination mandates when they are shared by a co-partisan politician. This section presents the results of testing this hypothesis. In particular, Table 4 shows the results from estimating Eq. 2. To identify the within-party co-partisanship effect, we run the model among Republican-leaning (Models 1 and 2) and Democratic-leaning counties (Models 2 and 3) separately. Furthermore, Fig 4 displays the predictive margins and the marginal effects of the model.

**Table 4:** Co-partisanship, communal values, and vaccination compliance.

Variable	Sample of counties			
	Safe Republican		Safe Democratic	
	Model (1)	Model (2)	Model (3)	Model (4)
Co-partisanship	3.464**	3.427**	3.061**	0.341
	(1.634)	(1.540)	(1.207)	(1.091)
Communal values		−0.982***		0.361
		(0.360)		(1.134)
Co-partisanship × Communal values		0.765*		−3.068**
		(0.403)		(1.448)
Constant	−2852.035***	−2395.526***	3943.548**	3775.101*
	(714.604)	(869.486)	(1754.070)	(2197.018)
Observations	818456	531388	145041	118589
R-squared	0.816	0.837	0.862	0.881
Health and economic controls	Yes	Yes	Yes	Yes

Notes: The dependent variable is Fully vaccinated people (%). Models 1-4 include state and month fixed effects. All models use two-way robust standard errors clustered by state and week. Models 1 and 2 are conducted over a sub-sample of 2000-20 stronghold Republican counties, whereas models 3 and 4 are conducted over a sub-sample of stronghold Democrat counties. Communal values refers to the absolute importance of communal moral values (2015-2018). Robust standard errors in parentheses. *** *p* < 0.01, ** *p* < 0.05, * *p* < 0.1.

When considering Republican counties, the presence of a co-partisan state governor is associated with a 3.5% higher vaccination rate, confirming the hypothesis of a positive co-partisanship influence on policy compliance. Moreover, this effect seems to be stronger when moderated by high communal values. As in-group loyalty and respect for authority are the predominant features in people sharing communal values, these values might magnify the already positive co-partisanship effect. A similar effect is also observed in Democratic-leaning communities, where the presence of a Democratic governor increases the total vaccination rate in the county by 3% on average. However, once we allow co-partisanship and communal values to interact, the relationship shifts and becomes negative. To conclude, while we establish that the unconditional co-partisanship effect on vaccination compliance is generally positive, we also demonstrate that its influence can be amplified or diminished depending on the prevalence of communal values within a county. Our results align with findings that show the effectiveness of messages from co-partisanship politicians in reducing vaccination non-compliance in the US [16,17,34].

### Robustness checks

To confirm the role played by political partisanship and emphasize the differences observed across US counties, we run six batteries of robustness checks to control for potential omitted variable bias, heterogeneity in the start of vaccination campaigns in US counties, potential endogeneity bias arising from the political partisanship measures we employed, multicollinearity between political party affiliation and communal moral values, false discovery rate (FDR), and geographic variations in media influence.

Despite the inclusion of a large array of control variables widely employed in similar studies, our political measures might still embody the effect of unobserved time-invariant county characteristics. To control for this possibility, we replicate the baseline analysis while adding the inclusion of county fixed effects, which, together with *month* and state×month fixed effects, assure that we are capturing not only the effect of monthly common and state-specific shocks but also that of county-specific features. As a second robustness check, we test whether differences in the early implementation of the vaccination campaign are actually driving the results. Indeed, the rollout of the vaccination campaign was uneven both across and within U.S. states, due to factors such as delays in vaccine supply and distribution challenges [59]. To control for this time heterogeneity, we replicate our baseline estimation after having time-centered and normalized the data around the date of vaccine introduction. The unit of observation remains the county. We next examine the potential endogeneity of political preferences. In particular, we re-construct the four partisanship measures after having excluded the data from the 2020 presidential election. This approach allows us to avoid the risk of endogeneity coming from political preferences toward candidates that were openly vaccine skeptical.

We also test for multicollinearity between political party affiliation and communal moral values, as it can undermine the statistical significance of our independent variable [60]. Specifically, we run eight regression models based on the baseline regressions in Table 2, incorporating both partisanship and communal value variables. A variance inflation factor (VIF) test is then conducted for each model to assess the extent to which multicollinearity inflates the variance of regression coefficients [61]. A VIF value exceeding 10 is commonly viewed as a sign that multicollinearity may be significantly affecting the least squares estimates [62]. We then control for the false discovery rate (FDR), which represents the expected proportion of rejections that are Type I errors (false rejections). To achieve this, we apply a two-stage procedure that estimates the number of true hypotheses to achieve sharpened FDR control and calculate Anderson’s sharpened q-values [63,64]. In this context, the q-value is interpreted as the probability that a significant test will result in a false positive. These q-values are then calculated for the coefficients of interest in the models presented in Tables 2, 4, and 5, which test Hypotheses 1 through 3.

Another important consideration is the role played by information availability and consumption during the vaccination campaign. (For example, studies indicate that although partisans tend to have relatively balanced media diets, their political attentiveness and media consumption fluctuate depending on whether news events are congenial to their party [65].) For instance, during the early phases of the vaccine rollout, public discourse was marked by significant uncertainty regarding antibody-dependent enhancement, original antigenic sin, and the effectiveness of blood-based vaccines in preventing mucosal infections. Variations in media coverage intensity on these issues could have shaped public attitudes toward vaccination and, in turn, influenced our findings. To address this concern, we replicate the baseline analysis, adding commutingzone×quarter fixed effects. Commuting zones provide more demographic and economic homogeneity than counties [66,67] and research shows that media consumption and exposure are heavily dependent on individuals’ location and social context, with certain areas more influenced by specific information channels [68,69]. While this study does not isolate how specific information influenced individual vaccination decisions, this robustness check accounts for location- and social context-dependent variations in information exposure during the rollout.

The results of the first three robustness checks and the final robustness check are presented in Tables S3 to S5 and Table S8. The outcomes of all four sensitivity analyses align closely with the baseline estimation, confirming the robustness of our initial findings. Results from the VIF test, shown in S6 Table, indicate that our independent variables do not exhibit significant multicollinearity issues. Finally, the S7 Table presents the sharpened q-values for the coefficients of interest in testing Hypotheses 1 through 3. With the exception of the co-partisanship coefficient in Table 5, Model 4 (q-value = 7.1%), all other coefficients maintain q-values below the 5%.

## Discussion

This paper examines the association between vaccine acceptance and political preferences in US counties during the vaccination campaign targeting SARS-CoV-2, and how this association is linked to the distribution of moral values, heterogeneity within parties, and co-partisanship effects. Given the complexity of these dynamics and the limited theoretical frameworks in the existing literature, this study adopts an exploratory approach to investigate these relationships.

First, we contribute to a growing literature on political preferences and attitudes toward vaccination by examining the heterogeneity underlying this relationship. In particular, we show that it is not only a county’s current party affiliation that matters, but also the duration of its affiliation. Accounting for this temporal dimension reveals differences in vaccination rates of around 1.5 percentage points. Furthermore, we demonstrate that compliance with public health measures is not uniform even within the same party: significant variation exists. For example, among Republican-leaning counties, vaccination rates differed by as much as 5.8 percentage points depending on the level of support for specific political leaders. This pattern is consistent with prior studies that highlight a link between support for Trump—beyond general Republican partisanship—and compliance with COVID-19 safety measures [23,24]. Interestingly, the divide around vaccination grew when the spread of the disease (and the fear of the virus) slowed from its peak during the second half of 2021. Taken together, these results suggest that using coarse, binary measures of political affiliation may overlook meaningful heterogeneity in public health behavior.

In addition to documenting heterogeneity, we also investigate underlying mechanisms, with a particular focus on why blue states have consistently achieved higher vaccination rates than red states. We find that a significant part of the effect of political partisanship on vaccination rates fades away when we account for moral values. This finding complements prior research studying how moral values mediate the relationship between political ideology and specific behaviors [70,71]. In particular, previous work has demonstrated that moral concerns about the binding foundations mediate the relationship between political orientation and acceptance of technological innovations. This occurs because when people evaluate innovations they draw from their moral values, which vary significantly between conservatives and liberals [70]. Our work extends this by demonstrating how moral judgments are linked to varying levels of adoption of a specific technology: vaccines. Furthermore, while the literature emphasizes the role of social capital in explaining compliance with public health measures [72,73], we show that the individual dimension (moral values) is more salient than the collective one (social capital).

Third, we also provide evidence of a positive co-partisanship effect, by showing that counties comply more with vaccination mandates when they are part of a state led by a co-partisan, i.e., where the governor is affiliated with the party that won the county in the presidential election. This holds both for blue and red counties, but while the diffusion of communal values reinforces the co-partisanship effect in Republican strongholds, the effect is the opposite in Democratic-leaning areas. This result highlights how Americans’ attitudes toward vaccination change based on who is in power. If the governor managing the vaccination campaign is a co-partisan, the likelihood of indulging in vaccine compliance increases for both Republicans and Democrats. This evidence reflects analogous results on sentiments toward the government’s data surveillance [74].

Finally, when interpreting our results, it is important to recognize that the relationship between moral values and vaccination behavior may not solely reflect moral motivations. For example, although universalist values—such as care, equality, and justice—are often associated with higher vaccination rates [25], this does not necessarily imply that individuals in areas where these values are more prevalent comply with public health policies because of greater altruism. Instead, these values may serve as proxies for other factors, such as higher levels of public trust and stronger institutional conformity [75]. In this sense, adherence to public health recommendations may also function as a form of identity signaling, expressing alignment with expert consensus and mainstream institutions. Similarly, communal values might reflect broader cultural and institutional dynamics—such as skepticism of centralized authority or preference for personal autonomy—rather than purely individual moral reasoning. We encourage future research to investigate the causal impact of moral values on public health compliance, as well as how these effects interact with other dimensions, such as those mentioned above.

### Limitations

While our results are the first to highlight the heterogeneity in the effect of partisanship on vaccination rates and the driving factors of this association, there are a few limitations that are worth noting.

First, it is important to exercise prudence in interpreting our geospatial results. Specifically, the observation that our county-related findings can also apply at the individual level could be subject to the ecological fallacy [76]. We argue that interpreting county-level correlations between moral values and vaccination behavior at an individual level is justifiable, a stance that is consistent with previous research [25]. Indeed, this stance is supported by the alignment of our results with previous research conducted at the individual level [28,77]. Additionally, our analysis controlled for a broad range of alternative potential factors influencing these county-level associations. Related to this issue, caution also should be applied when generalizing our findings to other levels of spatial aggregation, that is, just because counties higher in Care tend to have high vaccination rates does not imply that the same association holds across less granular (e.g., states) or more granular (e.g., neighborhoods or towns) levels of analysis. (This potential lack of generalizability across spatial units has been termed the “modifiable serial unit problem” [25,78].) Previous research using geospatial analysis has been able to replicate the observed construct–outcome associations across different spatial layers [79], but this has not always been the case [80]. Second, our results may not generalize to other countries. A crucial context that made the testing of our predictions possible was the fact that vaccines have been abundantly available in the United States. This certainly was not the case for many other countries throughout the pandemic.

A third limitation to the generalization of our findings concerns the specific time frame of the study (January 2021 to May 2022). This period was marked by exceptional circumstances, including the state of federal Public Health Emergency, high uncertainty, and the early stages of the vaccination campaign. While political partisanship has been shown to influence vaccination attitudes in both pre-pandemic contexts [7,15] and during the later, less critical, phases of the COVID-19 vaccination campaign [81], the intensity of the emergency during this period may have amplified these dynamics. (For instance, the uncertainty and stress caused by COVID-19 resulted in an increased reliance on social media use, which in turn fueled an infodemic of misinformation and conspiracy theories, particularly around vaccines [82,83].) As a result, the influence of moral values, co-partisanship, and partisanship in non-emergency contexts remains unclear, and future research should investigate whether their impact diminishes or shifts in more stable, non-crisis situations. Another shortcoming of our study is the inability to fully capture variations in the stringency of county-level COVID-19 interventions due to data limitations. However, we address variation at a broader geographical level by including state-by-month and month-fixed effects in our regression models, which control for policy changes and the emergence of new vaccine-related information at both state and federal levels. This approach is important because in the United States the federal government sets health policy standards and the states are the principal governmental entity responsible for implementing and enforcing them [84,85]. Nonetheless, future research with more granular data could better address this limitation and further explore the relationship between partisanship and local-level interventions. Finally, the Moral Foundations Questionnaire data utilized in our research were gathered between 2012 and 2018 [86], which does not align temporally with the vaccination data collected from 2021 onwards. However, we emphasize that there is evidence that individual differences in adherence to moral concerns as well as regional differences in other psychological characteristics are relatively stable over time [30,86,87].

## Conclusion

Since the fall of 2022, federal health officials in the US have begun exploring future strategies for vaccination against SARS-CoV-2, including the potential implementation of annual revaccination campaigns with updated vaccines targeting circulating variants [88]. However, over two years after the start of the vaccination campaign, more than 50 million American adults still did not complete the initial vaccination series, placing them at a significantly higher risk of hospitalization and death [88]. While vaccination mandates can boost uptake, they are not always feasible. For example, in 2022, the US Supreme Court blocked Biden’s workplace vaccine mandate. Additionally, mandates may yield unintended negative consequences that warrant consideration, such as undermining public trust—critical for sustaining future vaccine acceptance [89]—and worsening political polarization, human rights concerns, inequities, and social well-being [90].

Therefore, understanding the roots of vaccine non-compliance and developing effective, non-mandatory strategies to address it are essential. This research demonstrates that the factors contributing to vaccine refusal extend beyond mere party orientation, an aspect that has been emphasized in previous studies. Significant in this context is the role of individual moral values and co-partisan politicians. We believe that these results might have implications for policymakers and can be informative to increase compliance for future vaccination campaigns. Rational arguments about the benefits or risks of new technologies, including vaccines, often fail when they conflict with individuals’ underlying moral foundations, as individuals tend to frame arguments from their own perspectives [70]. However, research shows that when arguments are aligned with the moral values of the target audience, opinions and attitudes can shift significantly [91], a strategy that has also proven effective in health communication during the COVID-19 pandemic [92]. In the context of our findings, this approach could be applied to conservative regions with lower vaccination uptake by appealing to values of loyalty, framing vaccination as a patriotic duty to protect fellow citizens [25]. Similarly, messages aimed at individuals with weaker care-based moral foundations could highlight the convenience and ease of vaccination, emphasizing its speed and simplicity [26].

Prior research also indicates that people find messages more convincing and are more likely to take action when they come from members of their own in-group [93]. Our results complement this by identifying a co-partisan effect associated with higher vaccination rates. This suggests that, in addition to tailoring health communication to a community’s moral values, delivering these messages through politically aligned figures could further amplify their impact. It is important to note that these strategies would have been the most effective during the early phase of the vaccination campaign when all US states expanded vaccine eligibility to residents aged 16 and over (see S1 Fig). However, in September 2021, the US government mandated vaccination for a large segment of the workforce, reducing the relevance of vaccine hesitancy, as compliance became mandatory regardless of individual reluctance.

## Supporting information

S1 Fig Timeline of key events in the US COVID-19 vaccination campaign. Source: KFF COVID-19 Vaccine Monitor.
(TIFF)

S1 File Appendix. Contains all supporting tables and text.
(PDF)

S2 File Data availability statement. (PDF)
