# Supplementary figures and images for: Behind political affiliation: How moral values, identity politics, and party loyalty have affected COVID-19 vaccination
Source: PLoS One. 2025 Sep 26;20(9):e0330881. doi: 10.1371/journal.pone.0330881 (PMC12469336; doi:10.1371/journal.pone.0330881)

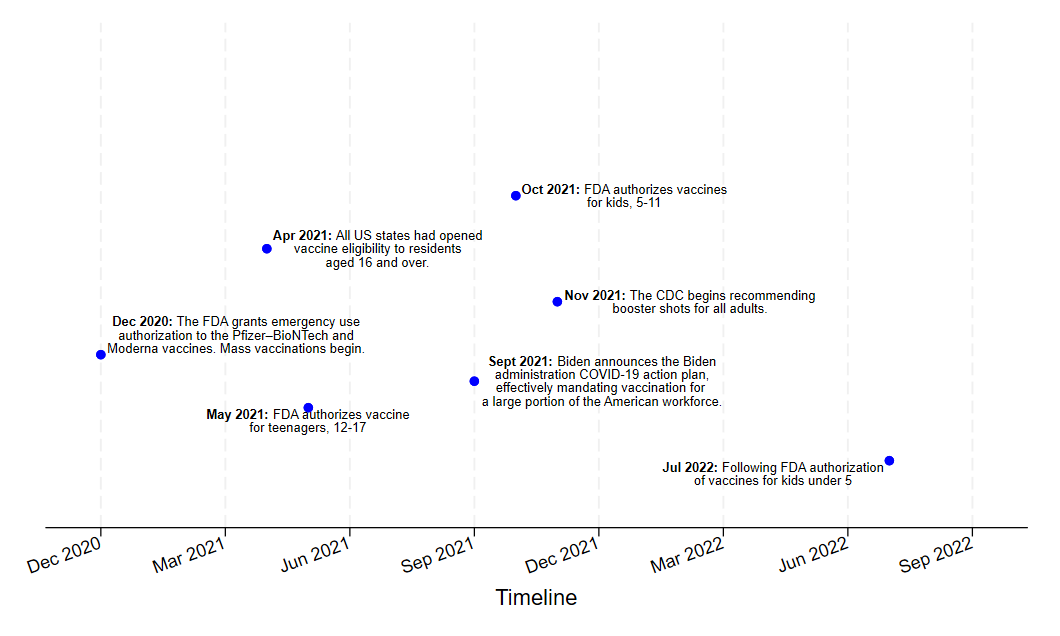

Supplement: S1 Fig — Source: KFF COVID-19 Vaccine Monitor. (TIFF) [file pone.0330881.s001.tif]
