# Supplementary material for: Behind political affiliation: How moral values, identity politics, and party loyalty have affected COVID-19 vaccination
Source: PLoS One. 2025 Sep 26;20(9):e0330881. doi: 10.1371/journal.pone.0330881 (PMC12469336; doi:10.1371/journal.pone.0330881)
Supplement: S1 File — Contains all supporting tables and text. (PDF) [file pone.0330881.s002.pdf]

Supporting information

S1 Table. Descriptive statistics

Variable	(1)<br>All counties<br>Mean (SD)	(2)<br>Democrat counties<br>Mean (SD)	(3)<br>Republican counties<br>Mean (SD)
Fully vaccinated people (%)	51.512<br>(12.181)	64.287<br>(13.483)	47.557<br>(9.854)
Rel. importance of communal vs. universalist values (2015-2018)	-0.064<br>(1.138)	-0.551<br>(0.824)	0.091<br>(1.187)
Social Capital Index	0.004<br>(1.004)	-0.573<br>(1.179)	0.076<br>(0.926)
Total case rate per 1000 people	251.789<br>(59.276)	243.306<br>(58.199)	253.542<br>(61.536)
Total death rate per 1000 people	3.813<br>(1.601)	3.095<br>(1.784)	4.063<br>(1.539)
Population density (people per square km)	106.276<br>(699.661)	490.415<br>(1775.506)	33.683<br>(61.383)
Percent of adults with less than a high school diploma, 2016-20	12.448<br>(6.038)	12.634<br>(7.159)	12.821<br>(5.711)
Percent of adults with a bachelor degree or higher, 2016-20	22.573<br>(9.702)	31.386<br>(14.143)	20.526<br>(7.394)
Unemployment rate 2021	4.613<br>(1.698)	5.853<br>(2.098)	4.304<br>(1.499)
Percent of people in poverty (2020)	13.751<br>(5.405)	15.588<br>(7.644)	13.622<br>(4.791)
Median household income (thousands $)	57.304<br>(14.499)	62.416<br>(22.019)	55.873<br>(12.437)
Prop HH with an internet subscription	33554.712<br>(103985.184)	118911.304<br>(228325.693)	15451.751<br>(28128.764)
Median age (years)	41.586<br>(5.440)	38.752<br>(5.389)	42.020<br>(5.209)
White population (%)	82.131<br>(16.419)	62.514<br>(22.531)	85.686<br>(11.819)
Black population (%)	9.125<br>(14.522)	22.873<br>(24.424)	6.769<br>(10.130)
Hispanic population (%)	9.617<br>(13.989)	15.220<br>(20.382)	8.757<br>(12.124)
HH with health insurance (%)	99.904<br>(0.050)	99.905<br>(0.057)	99.900<br>(0.049)
Observations	3099	448	2193

Notes: Average values are presented as mean coefficients. Values in parentheses indicate standard deviations.

S2 Table. The impact of relative communal values and high social capital on vaccination rates

**Variable**	Model (1)	Model (2)	Model (3)
Communal vs. universalist values × Q2 2021	-0.488*** (0.129)		-0.503*** (0.127)
Communal vs. universalist values × Q3 2021	-0.775*** (0.149)		-0.796*** (0.145)
Communal vs. universalist values × Q4 2021	-0.798*** (0.149)		-0.817*** (0.148)
Communal vs. universalist values × Q1 2022	-0.893*** (0.138)		-0.915*** (0.136)
Communal vs. universalist values × Q2 2022	-0.860*** (0.129)		-0.881*** (0.125)
Social Capital Index, upper quartile × Q2 2021		1.099*** (0.396)	2.023*** (0.460)
Social Capital Index, upper quartile × Q3 2021		0.735 (0.635)	2.607*** (0.660)
Social Capital Index, upper quartile × Q4 2021		-0.015 (0.815)	2.168*** (0.693)
Social Capital Index, upper quartile × Q1 2022		-0.178 (0.894)	2.491*** (0.782)
Social Capital Index, upper quartile × Q2 2022		-0.207 (0.871)	2.355*** (0.792)
Constant	38.465*** (1.637)	37.072*** (1.340)	37.758*** (1.577)
Observations	1025102	1574758	1025102
R-squared	0.959	0.952	0.959
County FE	Yes	Yes	Yes
State FE	No	No	No
Month FE	Yes	Yes	Yes
State x Month FE	Yes	Yes	Yes

Notes: The dependent variable is Fully vaccinated people (%). *Communal vs. universalist values* refers to Rel. importance of communal vs. universalist values (2015-2018). Models 1-4 include state, month and state*month fixed effects Q1, Q2, Q3, and Q4 refer to the periods: Jan 1–Mar 31, Apr 1–Jun 30, Jul 1–Sep 30, and Oct 1–Dec 31, respectively. All three models use two-way cluster robust standard errors (state and week). Robust standard errors are in parentheses. *** $p < 0.01$, ** $p < 0.05$, * $p < 0.1$.

S3 Table. Political partisanship and COVID-19 vaccination rates with county fixed effects

Variable	Model (1)	Model (2)	Model (3)	Model (4)
Partisanship measure	*Republican county*	*Partisanship in (2020)*	*Rooted High-Trump*	*Trump Support*
Republican county × Q2 2021	-3.963***<br>(0.787)			
Republican county × Q3 2021	-7.369***<br>(1.044)			
Republican county × Q4 2021	-9.135***<br>(0.956)			
Republican county × Q1 2022	-10.410***<br>(1.183)			
Republican county × Q2 2022	-10.819***<br>(1.245)			
Slightly Rep × Q2 2021		-1.839**<br>(0.763)		
Slightly Rep × Q3 2021		-3.507***<br>(1.093)		
Slightly Rep × Q4 2021		-4.179***<br>(1.002)		
Slightly Rep × Q1 2022		-4.318***<br>(1.119)		
Slightly Rep × Q2 2022		-4.493***<br>(1.064)		
Strongly Rep × Q2 2021		-4.410***<br>(0.896)		
Strongly Rep × Q3 2021		-8.236***<br>(1.222)		
Strongly Rep × Q4 2021		-10.203***<br>(1.115)		
Strongly Rep × Q1 2022		-11.626***<br>(1.375)		
Strongly Rep × Q2 2022		-12.125***<br>(1.440)		
Safe Rep × Q2 2021			-4.403***<br>(0.900)	
Safe Rep × Q3 2021			-8.110***<br>(1.266)	
Safe Rep × Q4 2021			-10.206***<br>(1.160)	
Safe Rep × Q1 2022			-11.574***<br>(1.452)	
Safe Rep × Q2 2022			-12.118***<br>(1.499)	
High-Trump-Support × Q2 2021				-2.062***<br>(0.482)
High-Trump-Support × Q3 2021				-3.983***<br>(0.695)
High-Trump-Support × Q4 2021				-5.116***<br>(0.445)
High-Trump-Support × Q1 2022				-5.793***

Continued on following page

				(0.439)
High-Trump-Support × Q2 2022				-6.114***
				(0.430)
Constant	40.644***	41.079***	40.910***	34.461***
	(1.443)	(1.450)	(1.497)	(1.294)
Observations	1574758	1574758	1574758	1293305
R-squared	0.956	0.957	0.957	0.957

Notes: The dependent variable is Fully vaccinated people (%). Models 1-4 include county, month and state*month fixed effects. Model 4 is conducted only on counties where Republicans obtained the majority of votes in the 2020 presidential election. Q1, Q2, Q3, and Q4 refer to the periods: Jan 1–Mar 31, Apr 1–Jun 30, Jul 1–Sep 30, and Oct 1–Dec 31, respectively. All models use two-way robust standard errors clustered by state and week. Robust standard errors are in parentheses. *** $p < 0.01$, ** $p < 0.05$, * $p < 0.1$.

S4 Table. Political partisanship and COVID-19 vaccination rates with time-centered vaccination data

Variable	Model (1)	Model (2)	Model (3)	Model (4)
Republican county	2.938***<br>(0.523)			
Republican county × Q2 2021	-4.575***<br>(0.723)			
Republican county × Q3 2021	-8.397***<br>(0.749)			
Republican county × Q4 2021	-9.750***<br>(0.880)			
Republican county × Q1 2022	-10.833***<br>(1.044)			
Republican county × Q2 2022	-11.208***<br>(1.092)			
Slightly Dem		1.457**<br>(0.589)		
Slightly Rep		1.680***<br>(0.466)		
Strongly Rep		2.881***<br>(0.589)		
Slightly Rep × Q2 2021		-2.511***<br>(0.687)		
Slightly Rep × Q3 2021		-4.690***<br>(0.906)		
Slightly Rep × Q4 2021		-5.264***<br>(1.003)		
Slightly Rep × Q1 2022		-5.437***<br>(1.125)		
Slightly Rep × Q2 2022		-5.558***<br>(1.116)		
Strongly Rep × Q2 2021		-5.192***<br>(0.836)		
Strongly Rep × Q3 2021		-9.596***<br>(0.894)		
Strongly Rep × Q4 2021		-11.156***<br>(1.054)		
Strongly Rep × Q1 2022		-12.368***<br>(1.263)		
Strongly Rep × Q2 2022		-12.839***<br>(1.309)		
Swing			2.490***<br>(0.460)	
Safe Rep			2.656***<br>(0.612)	
Safe Rep × Q2 2021			-5.102***<br>(0.815)	
Safe Rep × Q3 2021			-9.320***<br>(0.927)	
Safe Rep × Q4 2021			-10.997***<br>(1.107)	
Safe Rep × Q1 2022			-12.188***	

Continued on following page

			(1.344)	
Safe Rep × Q2 2022			-12.718***<br>(1.381)	
High-Trump-Support				2.437***<br>(0.478)
High-Trump-Support × Q2 2021				-2.435***<br>(0.478)
High-Trump-Support × Q3 2021				-4.667***<br>(0.532)
High-Trump-Support × Q4 2021				-5.293***<br>(0.503)
High-Trump-Support × Q1 2022				-5.657***<br>(0.555)
High-Trump-Support × Q2 2022				-5.873***<br>(0.570)
Constant	-574.065<br>(1124.886)	-563.028<br>(1139.129)	-322.079<br>(1153.538)	-3151.793***<br>(779.662)
Observations	1162144.000	1162144.000	1162144.000	925894.000
R-squared	0.860	0.862	0.863	0.860
Health and economic controls	Yes	Yes	Yes	Yes

Notes: The dependent variable is Fully vaccinated people (%). Models 1-4 include state, month, and state*month fixed effects. Model 4 is conducted only on counties where Republicans obtained the majority of votes in the 2020 presidential election. Health and economic controls include: unemployment rate, poverty rate, median income, proportion of people with less than HS diploma, population density, proportion of people with internet subscription, proportion of people older than 65 years old, proportion of minorities, proportion of people with health insurance, and number of hospital beds of all types per 1000 people. Q1, Q2, Q3, and Q4 refer to the periods: Jan 1–Mar 31, Apr 1–Jun 30, Jul 1–Sep 30, and Oct 1–Dec 31, respectively. All models use two-way robust standard errors clustered by state and week. Robust standard errors are in parentheses. *** $p < 0.01$, ** $p < 0.05$, * $p < 0.1$.

S5 Table. Political partisanship (in 2016) and COVID-19 vaccination rates

**Variable**	Model (1)	Model (2)	Model (3)	Model (4)
Republican county (2016)	2.243***<br>(0.485)			
Republican county (2016) × Q2 2021	-4.174***<br>(0.710)			
Republican county (2016) × Q3 2021	-7.696***<br>(0.821)			
Republican county (2016) × Q4 2021	-9.431***<br>(0.809)			
Republican county (2016) × Q1 2022	-10.583***<br>(1.062)			
Republican county (2016) × Q2 2022	-11.019***<br>(1.132)			
Slightly Dem		1.038**<br>(0.452)		
Slightly Rep		2.030***<br>(0.589)		
Strongly Rep		2.702***<br>(0.572)		
Slightly Rep (2016) × Q2 2021		-1.622**<br>(0.712)		
Slightly Rep (2016) × Q3 2021		-3.364***<br>(1.053)		
Slightly Rep (2016) × Q4 2021		-5.012***<br>(0.961)		
Slightly Rep (2016) × Q1 2022		-5.548***<br>(1.242)		
Slightly Rep (2016) × Q2 2022		-5.977***<br>(1.186)		
Strongly Rep (2016) × Q2 2021		-4.517***<br>(0.834)		
Strongly Rep (2016) × Q3 2021		-8.485***<br>(1.050)		
Strongly Rep (2016) × Q4 2021		-10.579***<br>(1.014)		
Strongly Rep (2016) × Q1 2022		-11.935***<br>(1.333)		
Strongly Rep (2016) × Q2 2022		-12.545***<br>(1.392)		
Swing			2.220***<br>(0.415)	
Safe Rep			2.305***<br>(0.534)	
Safe Rep (2016) × Q2 2021			-4.635***<br>(0.837)	
Safe Rep (2016) × Q3 2021			-8.468***<br>(1.056)	
Safe Rep (2016) × Q4 2021			-10.558***<br>(1.023)	
Safe Rep (2016) × Q1 2022			-11.820***<br>(1.309)	

Continued on following page

Safe Rep (2016) × Q2 2022			-12.395***	
			(1.359)	
High-Trump-Support				2.130***
				(0.412)
High-Trump-Support (2016) × Q2 2021				-1.925***
				(0.467)
High-Trump-Support (2016) × Q3 2021				-3.860***
				(0.650)
High-Trump-Support (2016) × Q4 2021				-4.883***
				(0.489)
High-Trump-Support (2016) × Q1 2022				-5.422***
				(0.505)
High-Trump-Support (2016) × Q2 2022				-5.644***
				(0.516)
Constant	-210.978	-171.173	-76.159	-2658.925***
	(1065.324)	(1084.698)	(1068.334)	(734.277)
Observations	1259727	1259727	1259727	1010850
R-squared	0.889	0.888	0.890	0.891
Health and economic controls	Yes	Yes	Yes	Yes

Notes: The dependent variable is Fully vaccinated people (%). Models 1-4 include state, month, and state*month fixed effects. Model 4 is conducted only on counties where Republicans obtained the majority of votes in the 2016 presidential election. Health and economic controls include unemployment rate, poverty rate, median income, proportion of people with less than an HS diploma, population density, proportion of people with internet subscription, proportion of people older than 65 years old, proportion of minorities, proportion of people with health insurance, and number of hospital beds of all types per 1000 people. Q1, Q2, Q3, and Q4 refer to the periods: Jan 1–Mar 31, Apr 1–Jun 30, Jul 1–Sep 30, and Oct 1–Dec 31, respectively. All models use two-way robust standard errors clustered by state and week. Robust standard errors are in parentheses. *** $p < 0.01$, ** $p < 0.05$, * $p < 0.1$.

S6 Table. VIF for political and moral variables

Political Variable	Moral Values Variable	VIF (Political, Moral)
Republican county	Communal vs. universalist values	2.23, 1.12
	Communal values	2.22, 1.15
Partisanship (2020)	Communal vs. universalist values	2.44, 1.11
	Communal values	2.42, 1.15
Rooted Partisanship	Communal vs. universalist values	2.42, 1.11
	Communal values	2.41, 1.15
High-Trump-Support	Communal vs. universalist values	2.08, 1.10
	Communal values	2.08, 1.14

Notes: VIF values are calculated from regression models that regress the monthly percentage of fully vaccinated people on partisanship and moral value variables, along with health and economic control variables, and include quarter, state, and quarter*state fixed effects. Health and economic controls include: unemployment rate, poverty rate, median income, proportion of people with less than HS diploma, population density, proportion of people with internet subscription, proportion of people older than 65 years old, proportion of minorities, proportion of people with health insurance, and number of hospital beds of all types per 1000 people. Communal values refers to the absolute importance of communal moral values (2015-2018). Communal vs. universalist values refers to relative importance of communal vs. universalist values (2015-2018).

S7 Table. Sharpened q-values for primary regression model specifications

Variable	Table 2	Table 4				Table 5			
**Republican county**	Models 1 - 4	Model 1	Model 2	Model 3	Model 4	Model 1	Model 2	Model 3	Model 4
Q2 2021	0.001								
Q3 2021	0.001								
Q4 2021	0.001								
Q1 2022	0.001								
Q2 2022	0.001								
**Strongly Rep**									
Q2 2021	0.001								
Q3 2021	0.001								
Q4 2021	0.001								
Q1 2022	0.001								
Q2 2022	0.001								
**Safe Rep**									
Q2 2021	0.001	0.015	0.001						
Q3 2021	0.001	0.006	0.001						
Q4 2021	0.001	0.001	0.001						
Q1 2022	0.001	0.001	0.001						
Q2 2022	0.001	0.001	0.001						
**High-Trump-Support**									
Q2 2021	0.001			0.023	0.001				
Q3 2021	0.001			0.002	0.001				
Q4 2021	0.001			0.001	0.001				
Q1 2022	0.001			0.001	0.001				
Q2 2022	0.001			0.001	0.001				
Co-partisanship						0.009	0.008	0.004	0.071
Co-partisanship × Communal values							0.014		0.009

Notes: The table presents the sharpened two-stage q-values for the coefficients of interest across the main regression models testing Hypotheses 1 through 3. To account for multiple hypothesis testing, the false discovery rate (FDR) is controlled, which refers to the proportion of false rejections (Type I errors). In total, 46 q-values are calculated across 12 different models.

S8 Table. Political partisanship and COVID-19 vaccination rates with commuting zone fixed effects

**Variable**	Model (1)	Model (2)	Model (3)	Model (4)
Partisanship measure	*Republican county*	*Partisanship in (2020)*	*Rooted partisanship*	*High-Trump Support*
Republican county	2.272*** (0.386)			
Republican county× Q2 2021	-2.593*** (0.604)			
Republican county × Q3 2021	-4.685*** (0.740)			
Republican county × Q4 2021	-6.120*** (0.655)			
Republican county × Q1 2022	-6.854*** (0.719)			
Republican county × Q2 2022	-7.041*** (0.762)			
Slightly Rep × Q2 2021		-1.567** (0.664)		
Slightly Rep × Q3 2021		-2.739*** (0.895)		
Slightly Rep × Q4 2021		-3.127*** (0.871)		
Slightly Rep × Q1 2022		-3.033*** (0.985)		
Slightly Rep × Q2 2022		-2.932*** (1.018)		
Strongly Rep × Q2 2021		-2.929*** (0.727)		
Strongly Rep × Q3 2021		-5.297*** (0.912)		
Strongly Rep × Q4 2021		-7.012*** (0.784)		
Strongly Rep × Q1 2022		-7.885*** (0.875)		
Strongly Rep × Q2 2022		-8.122*** (0.930)		
Safe Rep × Q2 2021			-2.835*** (0.724)	
Safe Rep × Q3 2021			-5.016*** (0.932)	
Safe Rep × Q4 2021			-6.700*** (0.832)	
Safe Rep × Q1 2022			-7.428*** (0.975)	
Safe Rep × Q2 2022			-7.752*** (1.001)	
High-Trump-Support × Q2 2021				-1.833*** (0.469)
High-Trump-Support × Q3 2021				-3.452*** (0.635)
High-Trump-Support × Q4 2021				-4.322***

Continued on following page

				(0.565)
High-Trump-Support × Q1 2022				-4.539***
				(0.620)
High-Trump-Support × Q2 2022				-4.828***
				(0.601)
Constant	-263.149	-260.930	-134.940	-1806.539***
	(927.274)	(932.835)	(958.482)	(539.764)
Observations	1259727.000	1259727.000	1259727.000	1010850.000
R-squared	0.924	0.924	0.924	0.927

Notes: The dependent variable is Fully vaccinated people (%). Models 1-4 include state, month and commuting zone*quarter fixed effects. Model 4 is conducted only on counties where Republicans obtained the majority of votes in the 2020 presidential election. Q1, Q2, Q3, and Q4 refer to the periods: Jan 1–Mar 31, Apr 1–Jun 30, Jul 1–Sep 30, and Oct 1–Dec 31, respectively. Data on commuting zones are retrieved from the Economic Research Service, US Department of Agriculture (USDA). All models use two-way robust standard errors clustered by state and week. Robust standard errors are in parentheses. ***$p < 0.01$, **$p < 0.05$, *$p < 0.1$.

S1 Text. Moral Foundations Theory.

Moral Foundations Theory provides a framework for exploring relationships between attitudes and moral value systems [1, 2]. In particular, MFT is drawn on the idea that people's moral values can be partitioned into five "foundations":

1. Care/harm: measures the extent to which people care for the weak and attempt to keep others from harm.
2. Fairness/reciprocity: measures the importance of ideas relating to equality, justice, rights, and autonomy.
3. In-group/loyalty: measures people's emphasis on being loyal to the "in-group" (family, country) and the moral relevance of betrayal.
4. Authority/respect: measures the importance of respect for authority, tradition, and societal order.
5. Purity/sanctity: measures the importance of ideas related to purity, disgust, and traditional religious attitudes.

Following previous studies based on MFT [3], we construct our indicator of moral values as the simple difference between communal and universalist values:

$$\begin{aligned} \textit{Relative importance of communal values} &= \textit{Communal values} - \textit{Universalist values} \\ &= (\textit{In-group} + \textit{Authority}) - (\textit{Care} + \textit{Fairness}) \end{aligned} \tag{1}$$

Importantly, the harm/care and fairness/reciprocity dimensions correspond to universalist moral values. For example, the fairness principle requires that people be fair, not that they be fair only to their neighbors. On the other hand, in-group/loyalty and authority/respect are tied to certain groups or relationships. In what follows, the fifth foundation is ignored because "divine" values are not directly related to the distinction between universalist and communal ones.

References

1. Amin AB, Bednarczyk RA, Ray CE, Melchiori KJ, Graham J, Huntsinger JR, et al. Association of moral values with vaccine hesitancy. Nat Hum Behav. 2017;1(12):873–880.
2. Graham J, Haidt J, Koleva S, Motyl M, Iyer R, Wojcik SP, et al. Chapter two – moral foundations theory: The pragmatic validity of moral pluralism. In: Advances in Experimental Social Psychology. Vol. 47. Academic Press; 2013.
3. Enke B. Moral values and voting. Journal of Political Economics. 2020;128(10):3679–3729.
