# Supplementary material for: Behind political affiliation: How moral values, identity politics, and party loyalty have affected COVID-19 vaccination
Source: PLoS One. 2025 Sep 26;20(9):e0330881. doi: 10.1371/journal.pone.0330881 (PMC12469336; doi:10.1371/journal.pone.0330881)
Supplement: S2 File — (PDF) [file pone.0330881.s003.pdf]

Behind political affiliation: how moral values, identity politics, and party loyalty have affected COVID-19 vaccination

Data availability statement

- The Center for Disease Control and Prevention (CDC) provided daily county-level data on cumulative COVID-19 vaccination rates starting from late December 2020. Data were collected on the day 30/12/2022.
- The New York Times GitHub repository provided data on coronavirus cases and deaths in the US. https://github.com/nytimes/covid-19-data
- The Massachusetts Institute of Technology (MIT) Election Data and Science Lab provided data at the county level on the total votes collected by each political party in the US presidential election held between 2000 and 2020.
- Enke (2020) provided data on the moral value scores at the county level for the period 2015-2018.
- American Community Survey through the United States Department of Agriculture (USDA) provided socioeconomic data like education, employment, data on poverty and income levels.
- The American Community Survey (ACS) - U.S. Census Bureau provided demographic and other types of data like internet subscriptions, data on sex and race, population density and health insurance coverage.
- The US Congress Joint Economic Committee provided data on the Social Capital Index
- Data on hospital beds per 1000 people and intensive care unit (ICU) beds are sourced from the Healthcare Cost Report Information System (HCRIS) and an open hospital facilities dataset produced by Definitive Healthcare.

All datasets were merged using the five-digit County Federal Information Processing Standard (FIPS) code identifiers. The data and STATA code used to reproduce the results presented in this paper are publicly available in the following online repository. The full replication package, including all code for running the analysis, contains:

- Code for generating summary statistics and preliminary analysis figures (`code1_prelim_analysis`).
- Code for reproducing regression results and figures from the main analysis (`code2_stat_analysis`).
- Code for performing robustness checks (`code3_robust_checks`).

Any additional information required to reanalyze the data reported in this article is available from the corresponding author upon reasonable request.
